# Spectral Imaging for Intracranial Stents and Stent Lumen

**DOI:** 10.1371/journal.pone.0145999

**Published:** 2016-01-05

**Authors:** Chi-Lun Weng, Ying-Chi Tseng, David Yen-Ting Chen, Chi-Jen Chen, Hui-Ling Hsu

**Affiliations:** 1 Department of Radiology, Shuang Ho Hospital, Taipei Medical University, New Taipei City, Taiwan; 2 Department of Radiology, School of Medicine, College of Medicine, Taipei Medical University, Taipei, Taiwan; Osaka University Graduate School of Medicine, JAPAN

## Abstract

**Introduction:**

Application of computed tomography for monitoring intracranial stents is limited because of stent-related artifacts. Our purpose was to evaluate the effect of gemstone spectral imaging on the intracranial stent and stent lumen.

**Materials and Methods:**

In vitro, we scanned Enterprise stent phantom and a stent–cheese complex using the gemstone spectral imaging protocol. Follow-up gemstone spectral images of 15 consecutive patients with placement of Enterprise from January 2013 to September 2014 were also retrospectively reviewed. We used 70-keV, 140-keV, iodine (water), iodine (calcium), and iodine (hydroxyapatite) images to evaluate their effect on the intracranial stent and stent lumen. Two regions of interest were individually placed in stent lumen and adjacent brain tissue. Contrast-to-noise ratio was measured to determine image quality. The maximal diameter of stent markers was also measured to evaluate stent-related artifact. Two radiologists independently graded the visibility of the lumen at the maker location by using a 4-point scale. The mean of grading score, contrast/noise ratio and maximal diameter of stent markers were compared among all modes. All results were analyzed by SPSS version 20.

**Results:**

In vitro, iodine (water) images decreased metallic artifact of stent makers to the greatest degree. The most areas of cheese were observed on iodine (water) images. In vivo, iodine (water) images had the smallest average diameter of stent markers (0.33 ± 0.17mm; *P* < .05) and showed the highest mean grading score (2.94 ± 0.94; *P* < .05) and contrast/noise ratio of in-stent lumen (160.03 ±37.79; *P* < .05) among all the modes.

**Conclusion:**

Iodine (water) images can help reduce stent-related artifacts of Enterprise and enhance contrast of in-stent lumen. Spectral imaging may be considered a noninvasive modality for following-up patients with in-stent stenosis.

## Introduction

Intracranial stents are becoming a popular choice for the treatment of intracranial cerebrovascular disease, such as atherosclerotic stenosis, aneurysm, or acute stroke. In patients with intracranial atherosclerosis disease, atherosclerosis is a risk factor for ischemic stroke. Recurrent stroke occurred more frequently in patients with symptomatic intracranial stenosis, even after the aspirin therapy [1]. Angioplasty combined with stenting, rather than angioplasty alone, gradually became a primary interventional treatment [2]. Although the Stenting versus Aggressive Medical Management for Preventing Recurrent Stroke in Intracranial Stenosis trial suggested that antiplatelet therapy and treatment of risk factor are more beneficial than endovascular stenting when using Wingspan stent system [3], the development of a new intracranial stent may redefine the role of endovascular intervention in intracranial atherosclerosis disease in the future. Aneurysm coiling is increasingly becoming a safer alternative to surgical treatment for patients with aneurysms. However, aneurysm coiling sometimes needs assistance of stents. Intracranial stenting is often considered as an adjunct for guiding the blood flow in the parent vessel and rebuilding the vessel wall simultaneously. Because of the increasing use of intracranial stents in neurointervention, some studies have indicated that up to 25%–35% of patients with intracranial stents for atherosclerotic arterial stenoses had in-stent restenosis. In patients with intracranial stents for intracranial aneurysm, the incidence of in-stent stenosis is 7.8% [4, 5, 6]. The evaluation of in-stent lumen has become a major concern.

Although conventional digital subtraction angiography (DSA) remains the gold standard of follow-up imaging techniques, noninvasive and reproducible techniques, such as computed tomography angiography (CTA) or magnetic resonance angiography (MRA), are invariably required. The application of CTA for monitoring intracranial stents has been limited because of stent-related artifacts [7]. Polychromatic X-ray beams are the primary sources producing metallic artifacts because of energy averaging. Thus efforts should be directed to reduce metallic artifacts and improve the spatial resolution of an image as much as possible. Dual energy computed tomography (CT), as an advanced technology in CT scanners, helps reduce the metallic artifacts and enhance the spatial resolution.

Presently, a dual energy CT scanner simultaneously acquires 2 data sets by using 2 energy levels from 2 X-ray tubes or by fast dynamic switching in a single generator [8]. After reconstruction and processing processes, spectral imaging, including material decomposition and monochromatic representation of data, is obtained. Monochromatic imaging can help reduce the beam-hardening effect and achieve a high image contrast at low energy. Material decomposition images can highlight a specific material such as iodine, which is more noticeable on images.

The present study aimed to evaluate the effect of spectral imaging on intracranial stent and in-stent lumen in the stent phantom and patients with intracranial stents.

## Materials and Methods

### Stent phantom

**Fig 1:**
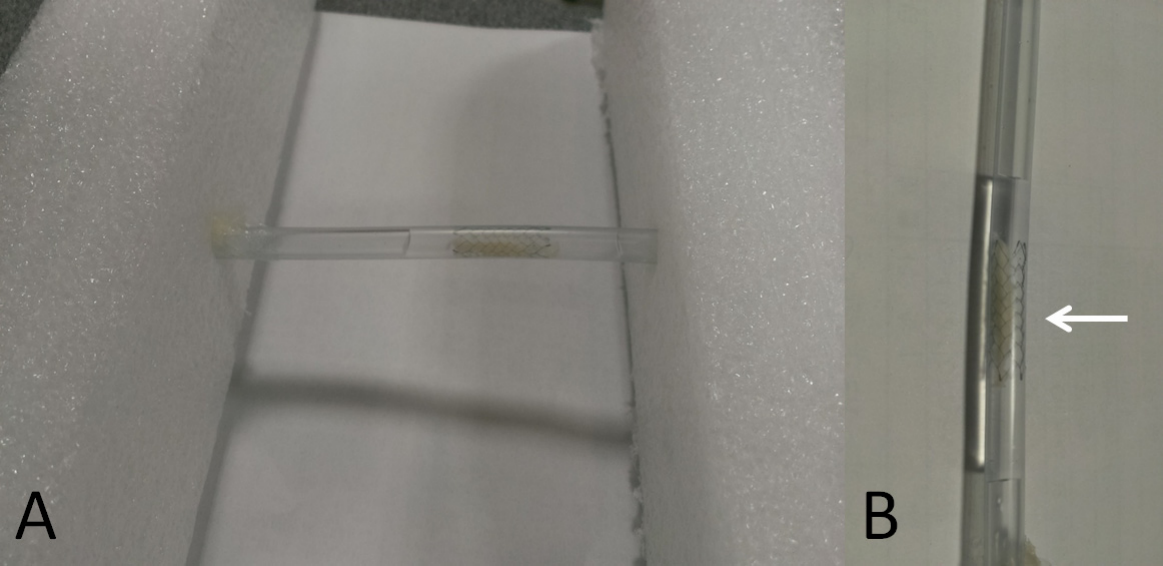
A stent phantom. (A) Photograph of a stent phantom. A phantom study was performed using a nitinol Enterprise stent, which was immersed in a nonionic iodinated contrast medium. (B) Close-up of the stent phantom. The white arrow indicates a lump of cheese in the stent.

We placed a self-expanding Enterprise stent (Raynham, MA, USA) into a silicon stent phantom, which was cylindrical and measured approximately 6.6 mm in diameter. The phantom was filled with 350 mg/mL of Iohexol (GE Healthcare, County Cork, Ireland), which was diluted with purified water to achieve an intraluminal density of 400 Hounsfield units, similar to that achieved in the brain CTA. The phantom was positioned on the table of a dual-energy CT scanner and scanned in the center of field of view. To simulate a plaque in a vessel, a lump of cheese was placed in the stent. We repeated the aforementioned process for the stent–cheese complex (Fig 1).

### Study population

From January 2013 to September 2014, 15 consecutive patients underwent Enterprise stent placement, and follow-up brain CTA with gemstone spectral imaging was obtained after 6–18 months, and CTA images were retrospectively reviewed. Overall, 18 intracranial stents were employed in these 15 patients. Although Enterprise stent was off-label use for patients with intracranial atherosclerotic stenosis or acute stroke, a study indicated that the rate of in-stent restenosis in patients with Enterprise stent for intracranial atherosclerotic stenosis had no significant difference from that in the Wingspan registry study. The proportion of symptomatic in-stent restenosis was lower in this Enterprise study (9.3%) than that in the Wingspan study (15.3%). Another advantage of Enterprise stent is more flexible than Wingspan stent, which makes Enterprise stent easier to reach every segment of circle of the Willis, even in tortuous vessels [5]. Above all, we used Enterprise stent to treat the patients with atherosclerotic lesion or acute stroke in spite of off-label use.

This study had been approved by Taipei Medical University-Joint Institutional Review Board with approval number: 201502045. The Taipei Medical University-Joint Institutional Review Board agreed to waive the informed consent because of the nature of this retrospective study. Patient records/information was anonymized and de-identified prior to analysis.

### Scanning protocol of brain CTA with gemstone spectral imaging

Brain CTA was performed using a Discovery CT750 HD 64-channel dual energy scanner (GE Healthcare, Milwaukee, Wisconsin, USA). The imaging parameters were set up for gemstone spectral imaging protocol, including a tube rotation of 0.6 seconds, slice thickness of 5 mm, fast energy switching between 70 and 140 kVp, tube current less than 640 mA, and pitch of 0.984. All images were reconstructed with 0.625-mm section thickness and 0.625-mm slice intervals. The reconstructed CT image data were sent to a workstation for generating material decomposition and monochromatic images by using gemstone spectral imaging viewer software (GE viewer 2.0 and GE Volume Share 4 AW 4.4, GE Healthcare) and displayed in axial view and multiplanar reconstruction.

### Evaluation of images

We used monochromatic 70-keV and 140-keV images and material decomposition images of iodine (water), iodine (calcium), and iodine (hydroxyapatite (HAP)) from spectral imaging to evaluate the effect of spectral imaging on the intracranial stent and in-stent lumen. Material decomposition images of iodine (water) means that the software further enhanced the density of iodine and suppressed water in the spectral imaging. Iodine (calcium) and iodine (HAP) both have the similar principle. HAP is a basic inorganic compound found in the skeleton and teeth of vertebrates and has a composition similar to that of a calcified plaque. We selected 70KeV and 140KeV images because the contrast noise ratio (CNR) of 70KeV images was similar to conventional CT and the 140KeV images reduced metallic artifact to the utmost degree on the monochromatic images. The window width and level were set to 1000 HU and 200 HU on 70-keV and 140-keV images, respectively, and to 260/90 HU, 150/−180 HU, and 300/−500 HU on iodine (water), iodine (HAP), and iodine (calcium) images, respectively.

Two radiologists (one with 10 years of experience in neurointervention; the other with 2 years of experience in neurology & head and neck) reviewed the spectral images on the workstation. The maximal diameter of radiopacity or dark portion of stent marker was measured using digital calipers in the axial view of each mode to evaluate the degree of decreased metallic artifact from stent markers. The maximal diameter was chosen by consensus of two radiologists.

For evaluation of CNR, two regions of interest of the same size and location were placed in each mode, one for the in-stent lumen and the other for adjacent brain tissue. The region of interest (ROI) of material decomposition images refers to density of iodine of the area and that of monochromatic images refers to CT number. CNR was defined as follows: CNR = (ROI_lumen_-ROI_brain_)/σ_brain_, where ROI_lumen and_ ROI_brain_ refer to iodine density or CT number in the in-stent lumen and adjacent brain tissue, respectively and σ_brain_ represented the standard deviation of ROI_brain_.

In addition, the two radiologists graded the visibility of the intraluminal stent area at the maker location using axial CT and multiplanar reconstruction images and a 4-point scale as follows: 1: The luminal area was completely invisible because of severe metallic artifacts or increased visual noise; 2: The visibility of the intraluminal stent area was between 0% and 25% caused by metallic artifacts or increased visual noises; 3: The visibility of the intraluminal stent area was between 25 and 50% caused by metallic artifacts or increased visual noises; 4: The visibility of the vessel lumen was more than 50%. The two radiologists outlined the visible luminal area at maker location manually and then calculated the percentage of visibility of lumen compared with lumen proximal to the marker location by using the software. A lower score indicated more severe stent-related artifacts or worse CNR. Disagreements between the radiologists were resolved by consensus to assign a final score.

The dose length product (DLP) of brain CTA with gemstone spectral imaging and dose area product (DAP) of diagnostic angiography of all 15 patients were recorded. The effective dose was calculated as follows: The effect dose of CT = DLP × dose conversion coefficient and that of angiography = DAP ×dose conversion coefficient. The dose conversion coefficient is 0.0026 mSv∙(mGy∙cm)^–1^ and 8.7×10^−4^ mSv∙(cGy∙cm^2^)^–1^ for brain in CTA and angiography, respectively.

### Statistical analysis

Statistical Product and Service Solutions version 20.0 (IBM Corp., Armonk, NY) was used to analyze all statistical data. All grading scores of each mode of gemstone spectral imaging, maximal diameter of the stent marker and CNR were finally calculated as the mean ± 1 standard deviation. The repeated measures analysis of variance was performed to evaluate the differences in quantitative scales. Multiple comparisons were performed by Bonferroni method and statistical significance was set at *P* < .05. Interobserver variation was evaluated using κ statistics.

## Results

### Stent phantom results

**Fig 2:**
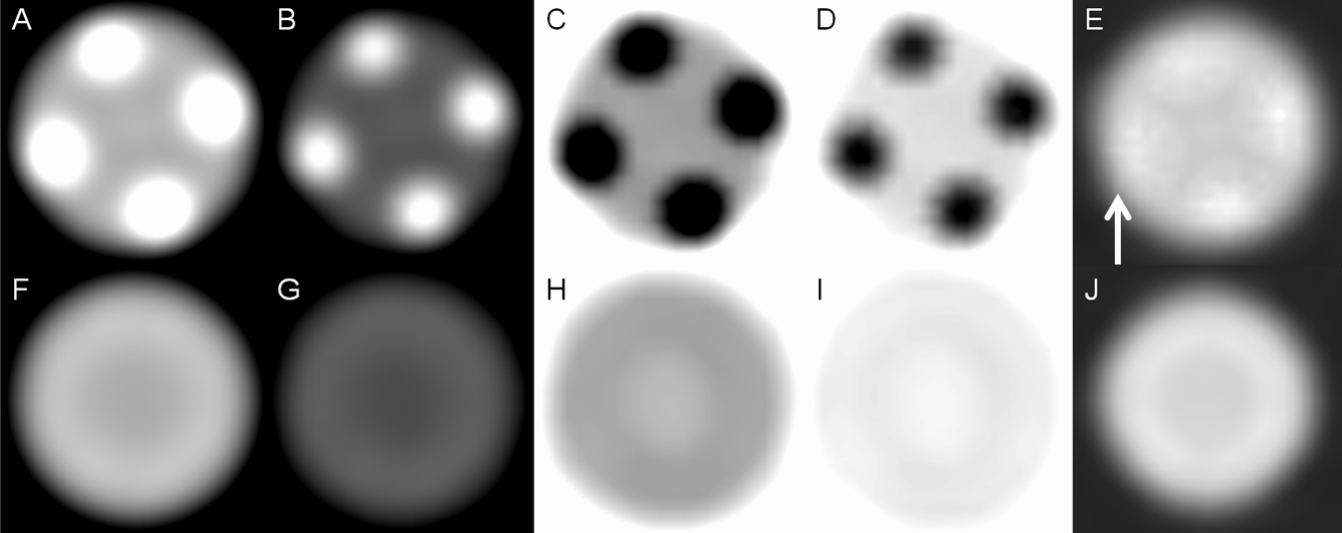
Stent phantom without cheese. (A) Monochromatic 70-keV, (B) monochromatic 140-keV, (C) iodine (calcium), (D) iodine (HAP), and (E) iodine (water) images of the marker location of the stent phantom show metallic artifacts on the iodine(water) images (white arrow) are considerably reduced. (F) Monochromatic 70-keV, (G) monochromatic 140-keV, (H) iodine (calcium), (I) iodine (HAP), and (J) iodine (water) images of the lumen location of the stent phantom reveal that nitinol produces few metallic artifacts in all modes.

**Fig 3:**
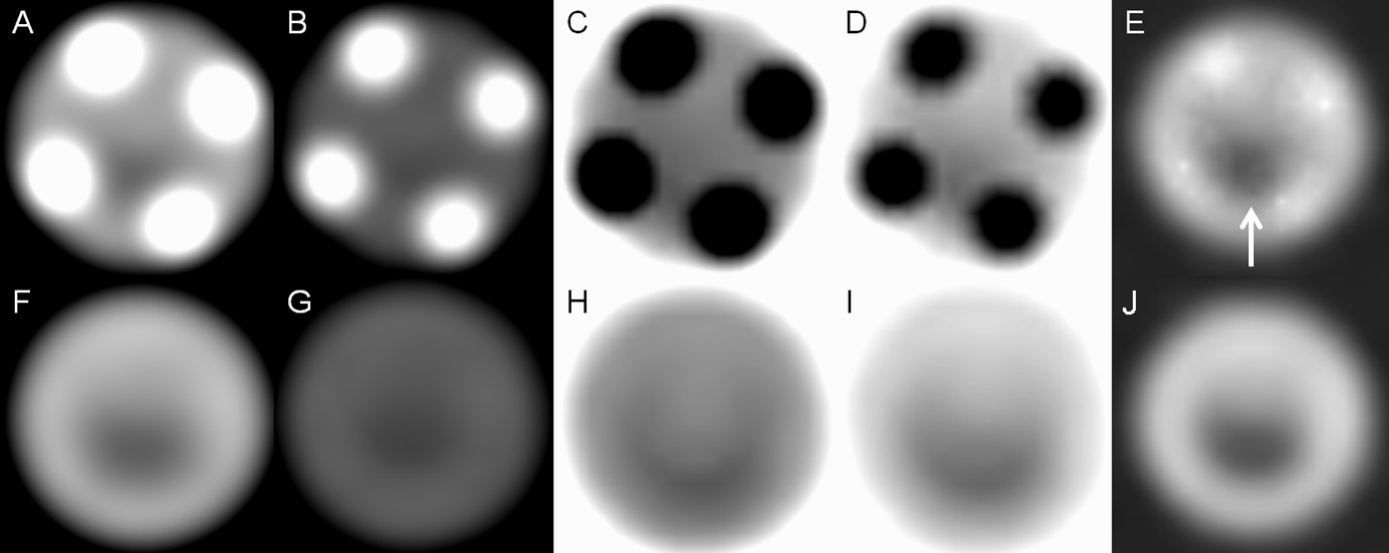
Stent phantom with cheese. (A) Monochromatic 70-keV, (B) monochromatic 140-keV, (C) iodine (calcium), (D) iodine (HAP), and (E) iodine (water) images of the marker location of the stent phantom show more areas of cheese (white arrow) on iodine (water) images because of decreased density of the radiopaque marker. (F) Monochromatic 70-keV, (G) monochromatic 140-keV, (H) iodine (calcium), (I) iodine (HAP), and (J) iodine (water) images of the lumen location of the stent phantom show that cheese can be more clearly observed on 70-keV and iodine (water) images.

When using the stent phantom, we observed that the radiopaque markers showed a mild blooming artifact on 70-keV images but the blooming artifact further decreased on 140-keV images. However, compared with 70-keV images, 140-keV images had low contrast. On iodine (water) images, the radiopaque marker showed few opaque densities, revealing more intraluminal areas covered by 4 radiopaque markers. Radiopaque markers became dark spots on iodine (calcium) and iodine (HAP) images. The mesh of the stent had few metallic artifacts on gemstone spectral imaging images. When using the stent–cheese complex, we obtained a satisfactory view of the cheese on 70-keV, iodine (HAP), and iodine (water) images. Because of decreased density of the radiopaque marker, more areas of cheese were observed on iodine (water) images at marker location. The cheese was partially obscured on 140-keV images and almost invisible on iodine (calcium) images (Figs 2 and 3).

### Patients’ results

**Fig 4:**
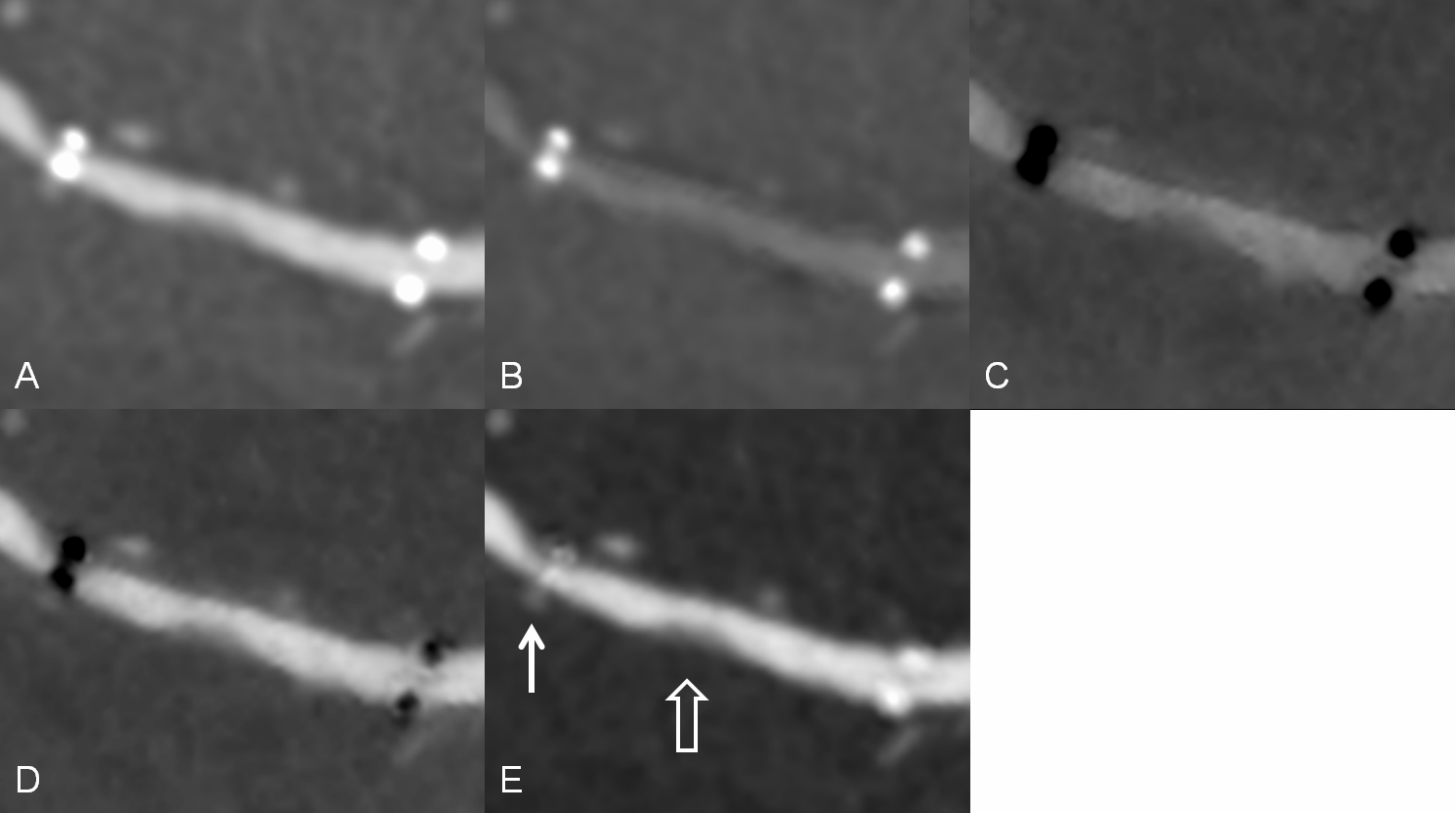
An intracranial stent in a patient. Multiplanar reconstruction of (A) monochromatic 70-keV image, (B) monochromatic 140-keV, (C) iodine (calcium), (D) iodine (HAP), and (E) iodine (water) images. The radiopacity of the stent marker was reduced in the iodine (water) images (white solid arrow). A soft plaque or intimal hyperplasia is visible in the in-stent lumen (white open arrow).

**Fig 5:**
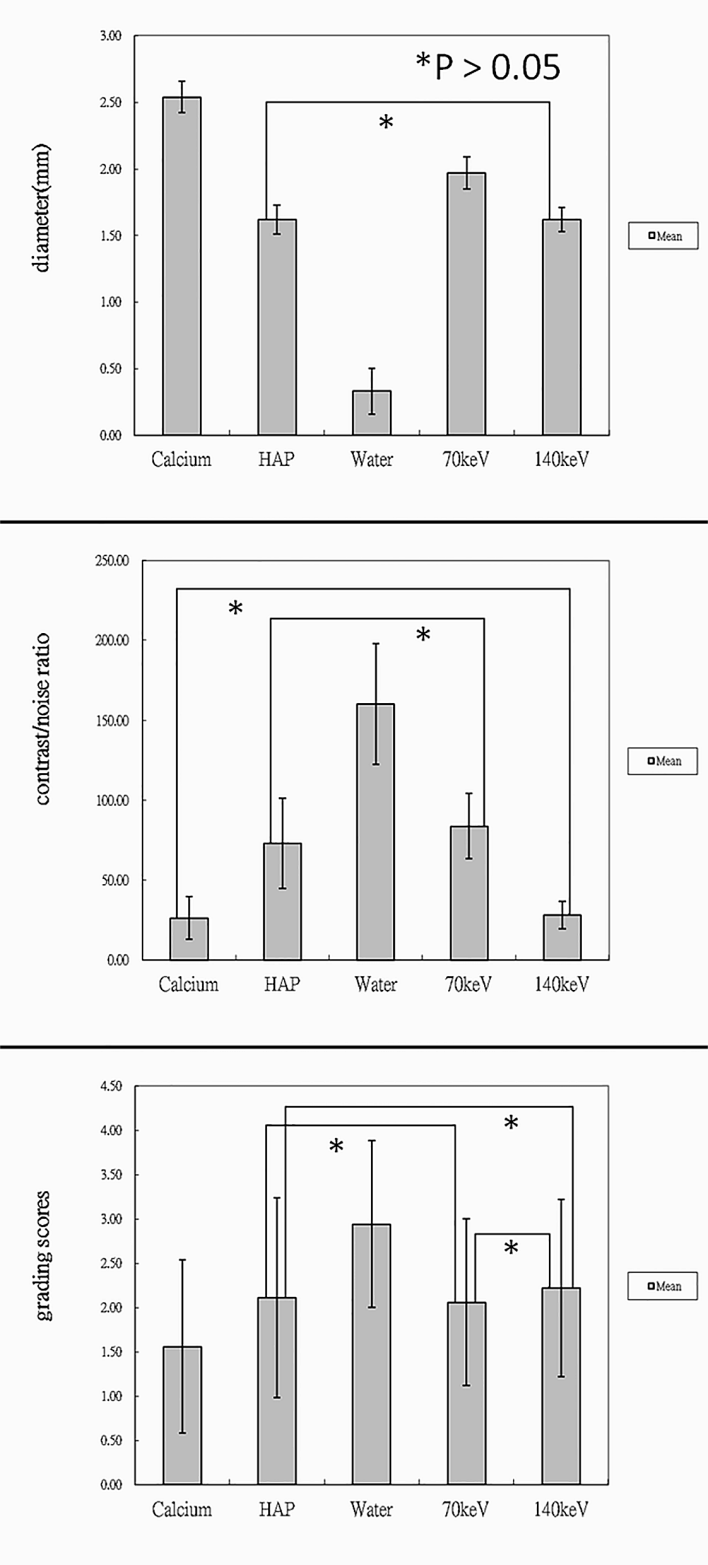
The mean of maximal diameter, contrast/noise ratio and grading score of visibility of intraluminal area at marker location. Three bar graphs showed that iodine (water) images markedly reduced radiopacity diameter of the stent marker, provided satisfactory image contrast and had the highest mean grading score, which was significantly different from those in the other modes.

The mean age of the 15 patients, including 11 men, was 60.8 years (range: 52–76 years). Eleven patients underwent intracranial stenting for tight stenosis, 3 for aneurysm, and 1 for acute stroke. In one of 3 patients for aneurysm, atherosclerotic stenosis and aneurysm simultaneously existed in the left MCA. The appearance of the radiopaque marker was similar to that of stent phantom (Fig 4). Iodine (water) images decreased metallic artifact of the stent maker most greatly with an average diameter measurement of 0.33 ± 0.17mm (*P* < .05). Iodine (calcium) images revealed the longest diameter of the stent marker (2.54 ± 0.12mm; *P* < .05). There were no significant differences in diameter measurement of the stent marker between iodine (HAP) and 140-keV images (1.62 ± 0.11mm versus 1.62 ± 0.09mm; *P* = 1.0). The mean diameter measurement on 70-keV images was 1.97 ± 0.12mm (*P* < .05) (Table 1, Fig 5).

**Table 1:** Effect of spectral imaging on the metallic artifact and contrast/noise ratio.

Modes of spectral imaging	diameter of stent marker	Contras/noise ratio
**Iodine (calcium)**	2.54 ± 0.12mm*^ƒ^	26.35 ± 13.4
**Iodine (HAP)**	1.62 ± 0.11mm	73 ± 28.02
**Iodine (water)**	0.33 ± 0.17mm^*ƒ*^	160.03 ±37.79^*ƒ*^
**70-keV**	1.97 ± 0.12mm^*ƒ*^	83.65± 20.47
**140-keV**	1.62 ± 0.09mm	28.13 ±8.38

*Mean ± standard deviation (all such values)

^ƒ^*P* < 0.05.

For CNR, iodine (water) images showed the highest average value (160.03 ±37.79; *P* < .05). In the other modes, the contrast/noise ratio, ranked in descending order was as follows: 70-keV images (83.65± 20.47), iodine (HAP) (73 ± 28.02), 140-keV images (28.13 ±8.38) and iodine (calcium) (26.35 ± 13.4). There were no significant differences when comparing 70-keV images to iodine (HAP) and 140-keV to iodine (calcium) (*P = 1*) (Table 1, Fig 5).

Regarding visibility of the intraluminal stent area at the marker location, iodine (water) images had the highest mean grading score of 2.94 ± 0.94 (*P* < .05). On iodine (water) images, 10 intracranial stents were graded 3 or 4 points (55.6%) and the other stents were graded 2 points (44.4%). By contrast, iodine (calcium) images had the lowest mean score of 1.56 ± 0.98 (*P* < .05). On iodine (calcium) images, most of the stents were graded 1 point (72.2%) and only 1 stent was graded 4 points (5.6%). In the other modes, the mean grading score, in a decreasing order, was 2.22 ± 1, 2.11 ± 1.13 and 2.06 ± 0.94 for 140-keV, iodine (HAP), and 70-keV images, respectively (Table 2, Fig 5). The mean grading score of iodine (HAP) at marker location did not show significant differences from that of 140-keV or 70-keV images (*P* = 1). There were also no statistically significant differences in grading scores between 140-keV and 70-keV images (*P* = 0.83). Interobserver agreement regarding the visibility of the intraluminal stent area at the marker location was high for each mode (κ = 0.768–0.841, *P* < .05).

**Table 2:** Patients’ profile and scores of visibility of in-stent lumen at marker location.

N	Age	gender	Iodine(calcium)	Iodine(HAP)	Iodine(water)	70KeV	140KeV	DLP of CT(mGy∙cm)	DAP of DSA(cGy∙cm^2^)	Stent location
1	76	M	4	4	4	4	4	1238.1	8453	Right cavernous ICA
2	67	F	1	1	2	1	1	833.33	8364	Left M1
3	65	F	1	3	4	2	3	1371.43	6462	Left M1
4	53	M	1	2	4	3	3	1266.67	7941	Right V4
5–1	65	M	1	1	2	1	1	1238.1	10741	Right V4
5–2	65	M	2	3	4	3	3	1238.1	10741	Left V4
6	59	F	1	1	3	1	1	1280.95	9614	Left MCA and ICA
7	52	M	1	1	2	1	1	1261.9	8725	BA and left V4
8	68	M	1	2	2	2	2	1280.95	9998	Left M1
9–1	55	M	1	2	3	1	2	1095.24	8795	Left MCA
9–2	55	M	3	4	4	3	4	1095.24	8795	Right MCA
10–1	60	M	1	1	3	2	2	947.62	7276	Left ICA
10–2	60	M	1	2	2	2	2	947.62	7276	Right M1
11	70	M	3	4	4	3	3	971.43	6381	Right M1
12	55	M	1	1	2	2	2	1280.95	8297	BA
13	56	M	3	3	4	3	3	957.14	9531	Right V4
14	56	M	1	2	2	2	2	1085.71	7263	Right M1
15	56	F	1	1	2	1	1	1290.48	9157	Left M1
Mean ± standard deviation			1.56 ± 0.98^ƒ^	2.11 ± 1.13	2.94 ± 0.94^ƒ^	2.06 ± 0.94	2.22 ± 1	1076.19	8487.13	

Iodine (water) images reveal the highest grading score of visibility of in-stent lumen at marker location. DLP = dose length product; DAP = dose area product; CT = computed tomography; DSA = digital subtraction angiography; ICA = internal carotid artery; MCA = middle cerebral artery; BA = basilar artery; M1 = 1^st^ segment of MCA; V4 = 4^th^ segment of vertebral artery.

^ƒ^*P* < 0.05.

The mean DLP of brain CTA with gemstone spectral imaging was 1076.19 mGy∙cm (833.33–1371.43 mGy∙cm) and the effective dose was 2.8 mSv. The mean DAP of previous diagnostic DSA was 8487.13 cGy∙cm^2^ (6381–10741 cGy∙cm^2^) and the effective dose was 7.38 mSv.

## Discussion

In 2013, Jin et al designed a prospective study to follow-up 226 patients with placement of intracranial stent for intracranial atherosclerosis disease. The authors demonstrated that patients with in-stent restenosis were more likely to have an early recurrent stroke than those without in-stent restenosis [9]. In another study, patients with intracranial atherosclerosis disease often had in-stent restenosis after placement of intracranial stents during follow-up [10]. In-stent stenosis also has been documented in the patients with stent for intracranial aneurysm treatment. Although these patients almost had no clinical symptoms during follow-up, symptomatic stenosis possibly occurred depending on length of stenotic lesion or stenotic rate [6]. Thus, physicians should routinely monitor for in-stent stenosis in patients with intracranial stent placement. However, patients often experience discomfort during a serial follow-up DSA and have a 0.79% risk of procedural complication [11]. According to previous studies, the angiographic follow-up rate was relatively low at approximately 28%–52% [4,12,13]. So far, 2 studies have suggested MRA by using quantitative or gadolinium-enhanced MRA for routine monitoring after intracranial stent placement [2,14]. Compared with MRA, conventional brain CTA is inexpensive, less time-consuming, and available for patients with contraindications to magnetic resonance imaging, such as a pacemaker or claustrophobia. However, stent-related artifacts degrade the image quality.

In the present study, iodine (water) images obtained through spectral imaging revealed that metallic artifacts caused by the stent marker were considerably reduced after decreased density of the radiopaque markers. These changes help radiologists to observe more in-stent lumen at the marker location. Material decomposition does not imply the degradation of materials. The basic principle of material decomposition is to form the attenuation coefficients of an unknown material using mass attenuation coefficients and effective densities of two basis materials in projection data by linear combination. The radiopaque marker of Enterprise is composed of tantalum covered by a polymer. Decreased density of the radiopaque marker on iodine (water) images is due to the combined effect of the tantalum’s x-ray attenuation curves of iodine and water. On iodine (calcium) and iodine (HAP) images, the radiopaque markers became dark objects, which are not helpful for reducing metallic artifacts. In addition, material decomposition images help enhance the presentation of the content of a specific basis material, such as iodine, to further improve tissue contrast. This property of the material decomposition images is advantageous for evaluating in-stent stenosis. The metallic artifacts caused by stent markers persistently exist on monochromatic images but are reduced on images obtained at high energy levels, although in-stent lumen can be partially visualized on monochromatic images. Overall, the iodine (water) mode provided satisfactory reduction of metallic artifacts caused by the stent marker and adequate contrast/noise ratio regardless of the lumen or marker location.

**Fig 6:**
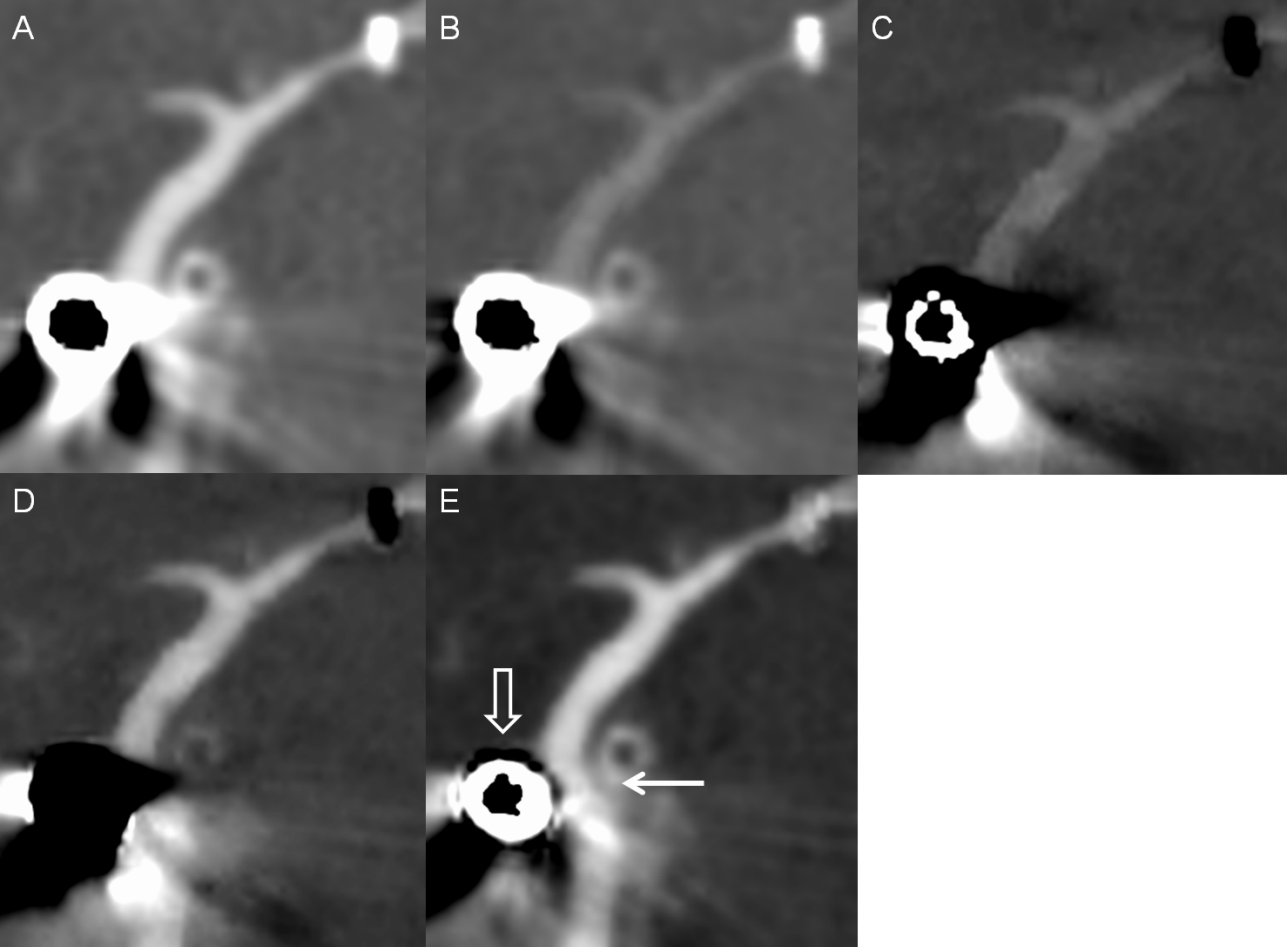
Intracranial stent and aneurysm coiling in a patient. Multiplanar reconstruction of (A) monochromatic 70-keV, (B) monochromatic 140-keV, (C) iodine (calcium), (D) iodine (HAP), and (E) iodine (water) images. The vessel lumen near the aneurysm coiling (white open arrow) is visible on the iodine (water) image (white solid arrow) and invisible on other images because of severe artifacts.

Two interesting findings are observed when using gemstone spectral imaging for evaluating intracranial stents. First, aneurysm coiling often produces severe metallic artifacts to obscure parent vessels on conventional CTA images. The present study included 3 patients with intracranial stents for assisting aneurysms coiling. On iodine (water) images, the artifacts caused by aneurysm coiling were substantially reduced. However, images obtained using the other modes did not show similar results. Fig 6 shows a typical case of reduction in the artifacts caused by aneurysm coiling. The reason remains unknown but reducing the artifacts caused by aneurysm coiling helps radiologists and physicians to easily observe the vessel lumen near the aneurysm. However, the number of cases is too small to have statistical significance. The effect of gemstone spectral imaging on aneurysm coiling needs further study.

**Fig 7:**
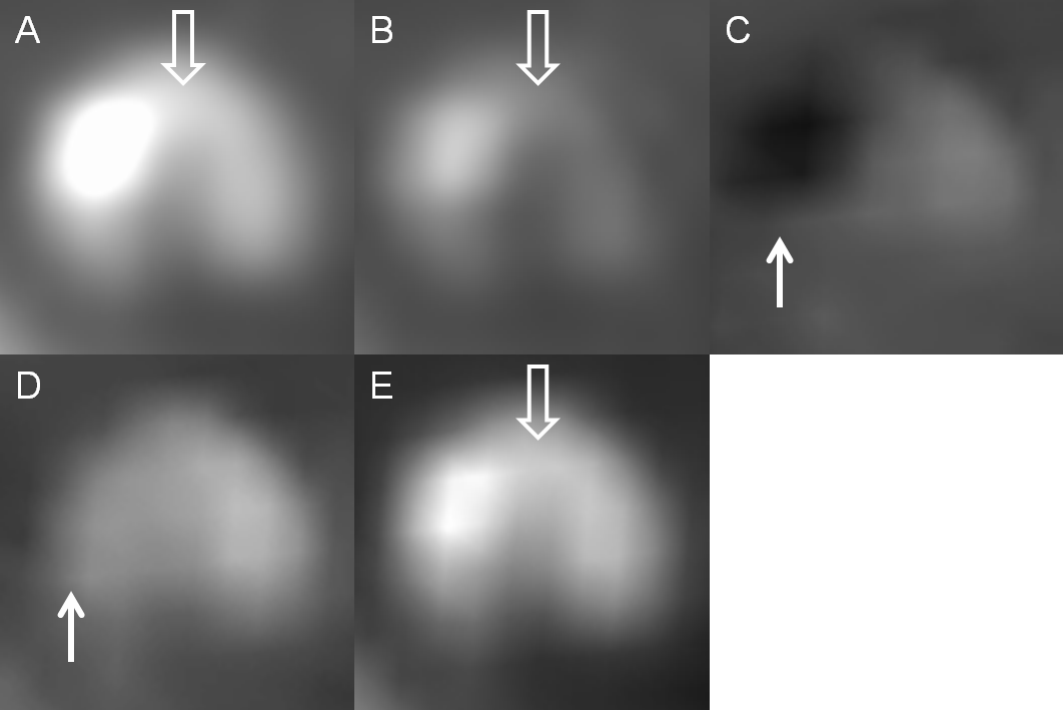
A mixed plaque in a patient with an intracranial stent. Axial view of (A) monochromatic 70-keV, (B) monochromatic 140-keV, (C) iodine (calcium), (D) iodine (HAP), and (E) iodine (water) images. The calcification of mixed plaque had the appearance similar to that of the stent marker and showed dark on iodine (calcium) images and disappeared on iodine (HAP) images (white solid arrow). Iodine (water) and 140-keV images reduced the blooming artifacts caused by the calcification of mixed plaque rather than change its hyperdensity. The noncalcified portion of mixed plaque remained hypodense on each mode and was visible on 70-keV, 140-keV and iodine (water) images (white open arrow).

Second, calcified plaques show a different appearance in each mode of gemstone spectral imaging. In this study, calcified plaques had a dark density similar to that of the stent marker on iodine (calcium) images but disappeared on iodine (HAP) images. This property of the plaques is sometimes helpful in distinguishing a calcified plaque and a stent marker. Iodine (water) and 140-keV images reduce the blooming artifact caused by a calcified plaque rather than change the high density of the calcified plaque. Noncalcified plaques remain hypodense in each mode and have a clear contrast to vessel lumen on iodine (water) and 70-keV images. The visibility of noncalcified plaques on 140-keV, iodine (calcium) and iodine (HAP) images was variable according to their component (Fig 7).

Radiation exposure is a major problem; in our study, the mean effective dose of brain CTA with gemstone spectral imaging was approximately 2.8 mSv, which was lower than that of the conventional brain CTA (4.88 mSv) at our institution during the same period and spectral imaging provided more information regarding the in-stent lumen combined with reducing the artifact simultaneously. Our study had some limitations. First, we included a small number of patients. Second, we studied only a single type of self-expanding nitinol stent, and results may vary with the use of different types of stents. Third, we do not have a gold standard to evaluate the accuracy of spectral imaging about in-stent restenosis.

In conclusion, iodine (water) images are beneficial for evaluating an in-stent lumen of Enterprise stent because they show markedly reduced metallic artifacts and provide satisfactory image contrast. Radiation exposure in brain CTA with gemstone spectral images is less than that in conventional brain CTA. We suggest that brain CTA with gemstone spectral imaging should be considered as a noninvasive modality for follow-up of in-stent stenosis.
